# 21^st^ century innovations in pediatrics

**DOI:** 10.3389/fped.2025.1700475

**Published:** 2025-11-20

**Authors:** Usha Ravi, Venkata Sushma Chamarthi, Saketh Parsi, Harikrishna Choudary Ponnam, Rahul Kashyap

**Affiliations:** 1 Tulare Pediatric Group, Pediatric Associates, Tulare, CA, United States; 2 Department of Pediatrics, Valley Children’s Healthcare, Madera, CA, United States; 3 Department of Medicine, Ascension Seton Medical Center, Austin, TX, United States; 4 Department of Medicine, Summa Health System, Akron, OH, United States; 5 Department of Research, Well-Span Health, York, PA, United States

**Keywords:** pediatrics, innovation, technology, telemedicine, artificial intelligence

## Abstract

21^st^-century innovations in pediatrics have made remarkable advancements in enhancing patient care and making care accessible even in remote areas. This abstract provides a concise summary of the latest innovations in pediatrics. Recent innovations in Pediatrics include Telemedicine, where people can connect with specialists using electronic technology, helping those in remote areas access timely care. Advances in neonatal care have effectively created solutions for several conditions, thereby reducing morbidity and mortality. The use of wearable technology has shown promise in several areas, including promoting a healthy lifestyle, monitoring physiological parameters, facilitating treatment for various conditions, and post-operative monitoring. AI in diagnostics is already aiding in earlier diagnosis and triage in emergency rooms, facilitating the diagnosis of certain genetic syndromes, and enabling the earlier detection of neonatal sepsis, among other areas in pediatrics. Innovations in pediatric vaccinations, utilizing novel technology, have made vaccines less painful and led to the development of new vaccines for conditions such as RSV, one of the viruses causing respiratory distress in newborns during the winter. Mental Health care in pediatrics has helped in reducing stigma and improving access to care for behavioral health counselling for conditions like depression and anxiety, using a smartphone app. Pediatric surgery has seen substantial progress in minimally invasive procedures, which help reduce post-operative pain, minimize tissue damage, decrease recovery time, and minimize scarring. Pediatric care now emphasizes addressing social determinants of health like housing, income, emotional support, food insecurity, and stressors in a child's life, which helps in timely referrals.

## 1 Background

In the 20th century, electronic health records (EHRs) brought about substantial changes in Pediatric primary care (PPC) workflow and the practitioner's experience. The late 20th century saw significant changes in the PPC workforce with the addition of nurse practitioners and physician assistants (1). The prevalence of attention deficit hyperactivity disorder, obesity, and mental health conditions rose (1). In the Neonatal ICU, with little care available for extremely premature infants in the early half of the 20th century, more intensive care was available, and increased survival rates were seen in the second half of the 20th century (2). Extremely preterm infants suffered respiratory distress due to a lack of pulmonary surfactant availability (2). In 1971, the world's first artificial medical consultant, INTERNIST-1, was created, which used a search algorithm to arrive at a clinical diagnosis based on patients' symptoms. It helped physicians to cross-check their differential diagnosis (3). Innovations in cell culture technologies in the 20th century enabled the development of effective vaccines to prevent polio and hepatitis A, as well as live attenuated vaccines to prevent polio (OPV), mumps, rubella, measles (MMR), rotavirus, and varicella (4). In this review, we discuss advancements made in the 21st century across various areas of pediatrics and aim to demonstrate the significant progress we have made since the previous century's status quo. A systematic search was not conducted due to the nature of the article being a narrative review.

### 1.1 Telemedicine in pediatric care

Telemedicine is a means of communication that utilizes electronic technology or telephones to enhance patient care. In the 21st century, Web 2.0 introduced more social networking and interoperability, and with the expansion of mobile technology, it has significantly helped physicians engage in real-time discussions with patients (5).

The main advantage of Telemedicine has been accessing care in rural and inner-city areas through specialist consultations. In addition, newborns and high-risk neonatal care in underserved areas is possible by contacting a sub-specialist elsewhere. It also has immense benefits in early screening by virtual appointments, seeing doctors from the comfort of the home, as well as timely access to pediatric care while being affordable (6).

In a study of telemedicine appointments among Asthma patients, the intervention group had significantly more intervention-free days compared to the control group (11.6 vs.10.7 days) (6). Patient satisfaction with telemedicine has shown promising results, where parents can manage their busy work schedules without taking time off to attend appointments at home (6).

During COVID-19 pandemic, telemedicine not only helped patients limit exposure to infection but also helped in conserving the personal protective equipment (6). More than 15 telemedicine programs are providing telemedicine consultations to patients in remote emergency departments across the United States (5).

Telemedicine is also being used for many pediatric sub-specialty appointments, where the number of in-person appointments can be limited. Pediatric gastroenterologists have been using it to manage chronic conditions, such as inflammatory bowel disease. Psychiatrists are seeing children for behavioral concerns using Telemedicine. A survey done in the University of California, San Francisco Pediatric Headache program for telemedicine follow-up visit showed 100% patients preferred telemedicine appointments and perceived it to be cost-effective, patient-centered and offering high patient and family satisfaction (5). A summary of these innovations are listed in Table 1.

**Table 1:** Brief presentation of 21st century innovations in pediatrics.

Telemedicine in Pediatric Care	Access to care in underserved areas Early screening by virtual appointments Cost-effective, patient centered.
Advances in Neonatal Care	Reduced invasive ventilation Stem cell therapy Epidermal electronic sensors Peripheral arterial oxygen monitoring.
Wearable Technology for Children	Increased physical activity Control of auxiliary disease Post-operative monitoring
Artificial Intelligence in Diagnostics	Predict risk of obesity and give dietary plans Early diagnosis and management Help triage in emergency rooms
Personalized Medicine in Pediatrics	Gender medicine-Tailoring treatment specific to gender Alternative therapies for diabetes, hypertension, cancer, epilepsy and mental health
Pediatric vaccination Innovations	Novel Technology Reverse vaccinology M-RNA vaccine technology
Mental Health in Pediatrics	Collaboration of Primary care with Psychiatrists Increased knowledge, prevention of suicide Many smart phone apps for counselling
Advances in Pediatric Surgery	Minimally invasive surgery Less post-operative pain Decreased scarring
Integration of Social Determinants of Health	Several programs support for at-risk children Family-centered risk assessments Timely referrals Access to community resources
Advances in Pediatric Oncology	CAR-T cell therapy Targeted treatment approach Immunotherapy
Digital Learning tools for Pediatric Rehabilitation	Tablet-based apps and VR devices Robotic devices Positive experience for children with special needs

### 1.2 Advances in neonatal care

In the 21st century, the use of technology to manage the delivery room environment has become popular. The International Liaison Committee on Resuscitation (ILCOR) committee was formed, which started making guidelines every 5 years to standardize neonatal resuscitation (7). At the onset of the new century in the year 2000, guidelines were made to use suction for meconium and 100% oxygen for resuscitation. Five years later, in 2005, recommendations were to use less suction for meconium and move to air for term resuscitation, and use T-piece devices and plastic bags. In 2010, the recommendation was to use a pulse oximeter to monitor heart rate and oxygen saturation, and to consider CPAP for respiratory distress, rather than using only 100% oxygen for resuscitation. In 2015, delayed cord clamping (DCC) was recommended for 30–60 s. In 2020, reducing invasive ventilation and using ECG to monitor heart rate were recommended. ILCOR is also recommended for thermoregulation with the use of warming adjuncts (warm blankets, plastic wrapping without drying, cap, thermal mattress), especially for infants <32 weeks old, to prevent hypothermia (<36 °C). DCC has been shown to reduce the risk of anemia, bleeding in the brain, and transfusions in premature babies (8).

In this new century, Stem cell therapy has emerged as a solution for many pediatric conditions that previously lacked effective treatment, including Hypoxic-ischemic encephalopathy (HIE), Necrotizing Enterocolitis (NEC), and Bronchopulmonary Dysplasia (BPD). Stem cell therapy, which utilizes mesenchymal cells (MSCs), is typically derived from bone marrow, placental tissue, and umbilical cord blood. It has neuroprotective effects, aids in tissue repair, and reduces inflammation (9).

Other innovations in neonatal care are peripheral arterial oxygen monitoring and epidermal electronic sensors (EES). The peripheral arterial oxygen monitoring is a non-contact method to help reduce the adverse effects associated with direct skin contact. One study showed that video-derived SpO2 signals obtained from estimating outputs of red and blue video channels could detect apnea by tracking decreases in oxygen saturations during apneic episodes over a long period (10). The EES has gained tremendous attention in the past 20 years due to its low cost. They are thin wearable sensors placed on the skin for extracting data on measurable physiologic parameters like body temperature, heart rate, stress, sweat composition, thus supporting clinical care, improving patient safety, and are of immense use in NICUs where continuous monitoring is vital (11).

### 1.3 Wearable technology for children

As sedentary lifestyles in children are associated with various health consequences, there is an increased promotion of wearables for preventive healthcare, control of auxiliary disease, and post-operative recovery monitoring (12). A systematic review, 18 out of 26 included studies focused on preventive care reported that wearable technology improves the physical activity of children and adolescents, which leads to a decreased sedentary lifestyle (12). Specific wearables have shown that they enhance the performance of children with cerebral palsy regarding physical activity (13).

Wearables can collect countless data with the patient being in the comfort of their home and facilitate data-driven management. Another study, which focused on tonsillectomy, found that wearables gave a more accurate estimation of step count compared to parent-reported logs (14). Bone-anchored hearing devices (BAHD) benefit kids with hearing loss, whether conductive or sensorineural, and have been used even with children who have ear malformations and a bone-conducting hearing device on a headband. It also aids children who are too young to undergo BAHD surgery (15). A study conducted in postoperative children with blood and bone marrow transplants found that data collected from wearables could be used by clinicians, caregivers, and patients to monitor symptoms and aid in developing personalized health strategies (16).

### 1.4 Artificial intelligence (AI) in diagnostics

Artificial Intelligence (AI) is enhancing preventive strategies by identifying risk factors and recommending measures to prevent a wide range of pediatric diseases. AI-based tools are predicting the risk of obesity in children by analyzing dietary habits, physical activity levels, and genetic predispositions, and even helping healthcare providers intervene early with personalized nutrition and exercise plans (17). AI has shown promising results in pediatric radiology, where machine learning algorithms can analyze medical images to detect conditions like pneumonia, brain tumors, and congenital heart defects with high accuracy. AI-based models have been developed to detect the risk of neonatal sepsis by analyzing electronic medical records and vital signs (18). Similarly, AI-driven algorithms can increase the likelihood of diagnosing developmental disorders like autism spectrum disorders by analyzing genetic and environmental data, leading to early diagnosis and management (18).

AI-enhanced socially assistive robots (SARs) address stress and discomfort in pediatric patients during painful emergency procedures. These robots utilize empathy, adaptive dialogue, and expressiveness during stressful times for children (19). Anti-microbial Prescription Surveillance Systems (APSS) aid in advanced clinical decision-making in pediatric emergency rooms by optimizing the transition from intravenous to oral antibiotics and improving treatment efficiency (19). AI's integration into pediatric emergency medicine is also helping tremendously with the issue of overcrowding and delays (19). AI is increasingly enhancing pediatric care by improving diagnostics, personalizing treatments, and streamlining clinical decision-making for better outcomes in children.

### 1.5 Personalized medicine in pediatrics

In the new era of medicine, modern innovative technologies have changed personalized medicine in pediatrics. A concept that involves analyzing the unique genetic code of individual pediatric patients and tailoring treatment approaches in a more precise and customized manner for better outcomes (20). Transformative changes in pediatric care, focusing on perinatal, neonatal, and pediatric critical care with a multitomic approach, have revolutionized the diagnosis of rare genetic disorders, thereby improving the prognosis and life expectancy (21).

Pediatrics has profited immensely from genomics discoveries, The tests available for over 2000 Mendelian conditions are changing the detection framework for rare genetic disorders. These methodologies have uncovered new insights into disease mechanisms and promoted alternative therapies for conditions such as diabetes, asthma, hypertension, cancer, epilepsy, blood disorders, and mental health (20). Personalized medicine in pediatric asthma and cardiac care has restructured treatment options, thus showing substantial benefits with fewer side effects (22). The growing concept of gender medicine, as a first step in personalized medicine, focuses on an integrated and comprehensive approach to treating diseases and achieving favorable outcomes (23). Factoring in individual differences and using genetic make-up information, personalized medicine has overhauled the paradigm of identifying and addressing many pediatric conditions into a whole-person approach.

### 1.6 Pediatric vaccination innovations

Vaccinations in pediatrics in the 21st century have flourished compared to those in the 20th century, largely due to the advancements in development technology. Novel Technology using virus-like particles has helped in vaccines against HPV. Reverse vaccinology has contributed to the recombinant vaccine against Meningococcus type-B (4).

Nirsevimab (Beyfortus), a monoclonal antibody, was approved by the FDA in July 2023 for preventing RSV-associated lower respiratory tract infections in infants under 24 months old (24). A 2024 study found that children and adults have a higher chance of surviving to their next birthday if they receive childhood vaccinations than if no vaccinations had occurred (25). mRNA vaccine technology, initially developed for COVID-19, is now being explored for pediatric infectious diseases (26). Vaccine innovations in the 21st century have greatly improved disease prevention and child health worldwide, enabling faster, safer, and more effective immunizations.

### 1.7 Mental health in pediatrics

In the past decade, it has become possible to support young people below 25 years with a variety of mental health conditions, as well as support their families with multidisciplinary and integrated health care (27). In the USA, the Massachusetts Child and Psychiatry Access Project (MCPAP) has created statewide collaborations between primary care practices and child and adolescent psychiatry services. MCPAP has a wide area of intervention, including ADHD, depression, and anxiety, as well as initiating pharmacological treatment, and studies have shown that young individuals enrolled in the program through primary care services have higher satisfaction and less risk of self-harm (27).

Behavioral health support is available to younger children as well as new parents through Bright Life in certain states of the U.S. (28). Some smartphone apps have been integrated with cognitive behavioral therapy to treat depression, anxiety, and other mental health conditions (29). An umbrella review showed that school-based targeted interventions like cognitive behavioral therapy have been shown to decrease symptoms of anxiety and depression. The school-based suicide prevention programs increased the knowledge of suicide and the prevention in the short term. Creative activities in the community have a positive effect on behavioral change and have also been shown to increase self-esteem and confidence (30). Increased awareness and focus on pediatric mental health have led to better tools for screening, diagnosing, and managing conditions like anxiety, ADHD, and depression. Digital mental health platforms provide accessible therapies, while school-based programs integrate mental health services into children's daily lives. These innovations reduce stigma and improve access to care.

### 1.8 Advances in pediatric surgery

In recent decades, pediatric surgery has achieved notable strides, owing to substantial progress in minimally invasive surgery (MIS). Returning to normalcy after MIS is much quicker and easier, because of smaller incisions and shorter hospital stays (31). By employing MIS in challenging pediatric procedures, pediatric surgeons can manage severely ill children of all age groups with less post-operative pain, decreased recovery time, reduction in tissue damage, and minimal scarring. Some recent advancements in MIS include; single-incision laparoscopy, robotic surgery, and endoscopy-assisted surgery (32).

Application and advances in tissue and organ bioprinting, regenerative medicine, and tissue bioengineering for fetal surgery have fueled a breakthrough in MIS (33). Due to controlled precision and undeniably improved outcomes compared to traditional open surgeries, MIS has become the preferred approach by many pediatric surgeons. However, limitations of training and resources have caused implementation hurdles in many parts of the world (34). With enormous benefits, MIS has pushed pediatric surgical boundaries to the next level.

### 1.9 Integration of social determinants of health

Pediatric care now emphasizes addressing social determinants, including housing, education, and nutrition, to improve long-term health outcomes (35). Social determinants of health (SDoH), such as safe housing, access to education, emotional support at home, food security, income, and physical activity, play pivotal roles in a child's daily growing environment and significantly impact how a child thrives. Many primary care clinics now include screening questionnaires for social factors as part of their well-child regular check-ups (36).

Early identification of social needs through various social risk-related screenings for hospitalized children and their families has improved health-related outcomes and the post-hospital discharge recovery phase within community-based organizations (37). General pediatricians can be instrumental in using various screening tools to identify home, school, and social stressors in a child's life and contribute significantly to improving and advocating for their overall well-being (38). Family-centered risk assessment, appropriate and timely referrals, and providing information and access to local community organizations and resources are the most effective interventions (39). A roadmap to a comprehensive approach to overcoming challenges in advancing healthcare equity and reducing healthcare expenditure is related to the social determinants of health (40).

### 1.10. Advances in pediatric oncology

The 21st century has brought targeted therapies for childhood cancers, such as CAR-T cell therapy for leukemia. Immunotherapy and precision medicine approaches are improving survival rates with fewer side effects. Advances in supportive care, like managing chemotherapy-related side effects, enhance the quality of life for pediatric oncology patients. There has been substantial progress in pediatric oncology diagnosis and treatment over the past decade, driven by advances in targeted therapies, liquid biopsies, functional precision medicine, and genomic profiling (41). New and improved technology has facilitated early detection and addressed the challenges of long-term post-treatment complications, as well as close monitoring for relapse (42).

Delving into the depths of tumor biology and heterogeneity helps us take a stride forward in developing new drug treatment combinations that improve outcomes of pediatric cancers. Newer strategies, such as CAR-T cell therapy and checkpoint inhibitors, are now being used for blood cell cancers and are being tested in solid tumors (42). Genomic profiling directed towards tumor mutations, combined with a more targeted treatment approach and immunotherapy, has been more effective and has fewer complications (43). It has been demonstrated in genetically guided clinical trials, such as “basket” and “umbrella” trials, where children are matched with their tumor genetics, rather than based on the location in the body. This represents a significant advancement in pediatric cancer treatment, particularly for rare, aggressive cancers where conventional therapies may be ineffective. The ongoing challenges for pediatric cancer drug development include, but are not limited to, strict regulations, limited patient numbers, restricted access to clinical trials, and hesitation to participate (44).

### 1.11. Digital learning tools for pediatric rehabilitation

An increasing number of applications with technological advancements not only support physical but also cognitive rehabilitation in the pediatric population (45). Telerehabilitation during the COVID-19 pandemic has made a significant positive impact on children with Specific Learning Disorders and Cerebral Palsy, with higher scale scores in learning, support, and respect categories (46). Several studies have been conducted with the Tach dino platform—a remote web-based platform for reading and spelling disorders- and have brought notable advantages for all children in need, despite their comorbidities (47).

Digital tools have not only transformed rehabilitative care but also made it more accessible and effective. Tablet-based apps and virtual reality (VR) not only attract the child to be more involved but also increase movement. For example, a study based on the FIT Board (Fun, Interactive Therapy Board) app is a tablet-based movement game app that brings together physical and e-learning (48). Another fun e-learning, speech therapy app for Autistic children that uses augmented reality is STAR (Speech Therapy with Augmented Reality), supports home-based speech therapy (49).

 There has been broad acceptance of digit-AI health tools such as mobile apps, VR devices, robotic devices, games, telehealth apps, and wearables by pediatric health care professionals in both hospital and rehab outpatient settings (50). Despite many factors that facilitate and restrict usage, clinicians support implementation and have greater benefits for children with special needs (50). Overall, digital health tools have transformed pediatric rehabilitative care by reorganizing and integrating modern technology, telemedicine, and promoting positive experiences in children with special needs.

## 2 Conclusion

In the 21st century, pediatric care has been transformed by innovative technologies and approaches. Telehealth and mental health apps have expanded access to counseling and support while remaining confidential. Precision medicine and minimally invasive surgeries have enhanced treatment outcomes and reduced recovery times. Additionally, AI is playing a growing role in emergency care by improving triage efficiency. These advancements collectively promise a more personalized, accessible, and effective pediatric healthcare system.

It is essential that pediatric professionals actively monitor and evaluate these technological advancements to ensure they align with high-quality and ethical patient care. Pediatricians play a very important role in assessing the appropriateness, safety, and effectiveness of new technologies before integrating them into clinical practice. As digital innovations continue to reshape pediatric medicine, ongoing professional education, collaboration with interdisciplinary teams, and adherence to established ethical guidelines can help clinicians make informed decisions that prioritize well-being, privacy, and dignity of children and families. Pediatric professionals can foster trust, safeguard patient rights, and ensure innovation serves as a complement rather than a replacement for compassionate, human-centered care.
